# Antibiotics in the medical and surgical treatment of necrotizing enterocolitis. A systematic review

**DOI:** 10.1186/s12887-022-03120-9

**Published:** 2022-01-27

**Authors:** Ester Maria Gill, Kristine Jung, Niels Qvist, Mark Bremholm Ellebæk

**Affiliations:** 1 Research Unit for Surgery, and Centre of Excellence in Gastrointestinal Diseases and Malformations in Infancy and Childhood (GAIN), Odense University Hospital, J.B. Winsløws Vej 4, 5000 Odense C, Denmark; 2 University of Southern Denmark, Odense, Denmark

**Keywords:** Necrotizing enterocolitis, Antibiotics, Surgery, Death

## Abstract

**Background:**

The aim of this review was to identify relevant randomized controlled trials (RCTs) and non-RCTs to evaluate the existing knowledge on the effect of antibiotic treatment for infants with necrotizing enterocolitis (NEC).

**Objective:**

Identifying 1) the best antibiotic regimen to avoid disease progression as assessed by surgery or death, 2) the best antibiotic regimen for infants operated for NEC as assessed by re-operation or death.

**Methods:**

Embase, MEDLINE and Cochrane were searched systematically for human studies using antibiotics for patients with NEC, Bell’s stage II and III.

**Results:**

Five studies were included, with a total of 375 infants. There were 2 RCT and 3 cohort studies. Four main antibiotic regimens appeared. Three with a combination of ampicillin + gentamycin (or similar) with an addition of 1) clindamycin 2) metronidazole or 3) enteral administration of gentamycin. One studied investigated cefotaxime + vancomycin. None of the included studies had a specific regimen for infants undergoing surgery.

**Conclusions:**

No sufficient evidence was found for any recommendation on the choice of antibiotics, the route of administration or the duration in infants treated for NEC with Bell’s stage II and III.

## Backgound

Estimated 1–3 of every 1000 newborns are affected by Necrotizing Enterocolitis (NEC) [1]. The majority (90%) of cases are seen in very low and extremely low birthweight (BW) infants, with a gestational age (GA) < 32 week [2]. Among the infants with the lowest BW and GA the incidence may be as high as 22% [2, 3]. The medical treatment consists of antibiotics and cessation of enteral feeding. The rationale is immaturity of the gastrointestinal tract and the innate immune system. In severe cases (Bell’s stage III) surgery may be needed [4] and the indication for postoperative antibiotic treatment is to prevent surgical site infections and recurrence of NEC.

The antibiotic treatment of NEC empirically covers anaerobic and Gram-negative bacteria. The most common regimen reported is intravenous administration of ampicillin and gentamycin combined with metronidazole for a period of 10–14 days [5, 6]. A Cochrane review from 2012 included randomized and quasi randomized studies and could not provide sufficient evidence for a general recommendation for a specific regimen of antibiotics in the treatment of NEC [7]. Several studies have investigated the effects of prophylactic antibiotics to prevent NEC in infants of risk but without any evidence for the effectiveness [8, 9]. Broad spectrum antibiotics covering both aerobic and anaerobic bacteria are recommended [10], because no single organism has proven to consistently cause the disease [11]. Cultures from blood/feces/operative specimen or vascular access devices in NEC-patients treated with a combination of ampicillin, gentamicin and metronidazole showed complete sensitivity to all strains in only 4 out of the 12 included patients [6].

Several reviews have tried to recapitulate the most common treatment combinations used but without a comparison of effectiveness [5, 7, 12]. The rarity and heterogenicity of the disease make it difficult or even impossible to perform clinically randomized trials (RCT) to achieve robust results,

The primary objective of the present study was to clarify which antibiotic regimen is the most effective for children with NEC to avoid progression of disease as assessed by operation or death.

The secondary objective was to identify the effect of antibiotic regimens in children, who have undergone surgery as assessed by the need for reoperation or death.

## Methods

This systematic review was performed in accordance with the PRISMA guidelines [13].

### Study registration

The study was registered in the PROSPERO register (CRD42020162193) January 15th, 2020.

### Eligibility criteria

Studies with documented NEC according to Bell’s classification stage II or III, treatment with at least one type of antibiotic and with reported progression of the disease defined as surgery and/or death as an outcome were included. Exclusion criteria were 1) inability to obtain the full text and insufficient data on primary outcomes, 2) studies with overlapping data, 3) case reports 4) non-original articles such as reviews 5) articles not available in English or Scandinavian languages (Danish, Norwegian or Swedish).

### Information sources and search strategy

The following databases were searched: Embase, Medline (both through Ovid) and Cochrane. Clinicaltrials.gov and Prospero were searched for any relevant ongoing trials or reviews. Opengrey.eu was searched to locate relevant grey literature within the area.

Relevant search terms were identified in Embase and Medline. The following search was conducted in Embase and adjusted to fit Medline and Cochrane format:

(prematurity [Emtree] or extremely low birthweight [Emtree] or very low birth weight [Emtree] or low birthweight [Emtree] or newborn [Emtree] or infant [Emtree] or Infant* or extremely premature infant*or premature infant* or premature neonate or pre-mature infant or pre-term bab* or pre-term child* or pre-term infant* or pre-term neonate or pre-term newborn or premature or premature bab* or premature child* or premature neonate* or premature newborn* or prematuritas or preterm bab* or preterm child* or preterm infant* or preterm neonate* or preterm newborn* or human neonate* or human newborn* or human infant* or Neonate* or Newborn child* or Newborn bab* or Neonatal Prematurity or Prematurity or Extremely Premature Infant* or Extremely Preterm Infant* or Extremely Premature Infant* or extremely low birthweight infant or extremely low birthweight infant or ELBW or very low birthweight infant or very low birth weight infant or VLBW or low birthweight infant or low birth weight infant or LBW) AND (necrotizing enterocolitis [Emtree] or necrotising enterocolitis or necrotizing enterocolis) AND (antiinfective agent [Emtree] or antibiotic agent [Emtree] or antibiotic prophylaxis [Emtree] or amoxycillin or amoxicillin or ampicillin or azithromycin or augmentin or benzylpenicillin or b-lactam or beta-lactam or beta-lactams or clarithromycin or cefuroxime or cotrimoxazole or co-trimoxazole or cefotaxime or ceftriaxone or ceftriaxone or cefditoren or chloramphenicol or cefpodoxime or cephradine or cephalexin or cefetamet or cefaclor or cephalosporin* or erythromycin or gentamicin or gentamycin or levofloxacin or minocycline or metronidazole or moxifloxacin or penicllin* or quinolone* or roxithromycin or sulphamethoxazole or sulfamethoxazole or trimethoprim or Antibiotic* or folate antagonists or combined antibiotic* or antibiotic or antibiotic prophylaxis or anti bacterial agent* or anti infective agent* or antibacterial agent* or antibacterial drug* or antibacterial spectrum or antiinfective* or antimicrobial* or antiseptic* or microbiological agent* or Anti-Bacterial agent* or Antibacterial Agent* or Anti-Bacterial Compound* or Anti Bacterial Compound* or Bacteriocidal Agent* or Bacteriocide* or Anti-Mycobacterial Agent* or Anti Mycobacterial Agent* or Antimycobacterial Agent*)The search was finalized by excluding conference abstracts from the search. A third search was performed by manually checking the references listed in the included studies. All relevant studies were included in the original search.

### Study selection

Covidence^14^ was used for the screening process. The initial screening was carried out by the authors EMG and KJ. Included studies were screening based on title and abstract. If conflicts occurred, reasons for exclusion were discussed until agreement. Full text screening was carried out by the same two authors. In case of conflict, the article was discussed until a mutual agreement among all authors were obtained.

### Data collection and data items

Data collection was conducted with a standardized piloted form within Covidence [14] and on an external platform allowing for extraction in duplicate by both authors. All extracted data was reviewed by both authors to ensure consistency in agreement. When information was missing from the included articles the author [15] was contacted.

The list of study items to be extracted was made according to the Cochrane handbook chapter 5 [16] and modified according to relevance for this review. Extracted items included: Author name, title, publication year, study method, number of included infants, type of antibiotics used, doses, frequency, and route of administration. The number of infants who died or underwent surgery was retrieved. For the group of patients that underwent surgery the postoperative use of antibiotics was registered and the number of postoperative deaths or reported reoperation for NEC was retrieved.

### Risk of bias in individual studies (methodological quality appraisal)

RCTs were analyzed for bias using the Risk of Bias tool RoB2 [17]. RoB2 evaluates the risk of bias in a study based on 5 domains of bias: 1) the randomization process 2) deviations from the intended intervention 3) missing outcome data 4) measurement of the outcome 5) selection of the reported result. Each domain is graded low, high, some concerns or no information according to a set of signaling questions, and later summed in an algorithm to determine bias level. The n-RCTs included were analyzed using ROBINS-I [18]. ROBINS-I evaluate how well the n-RCT study handles bias spread across 7 domains compared to a well performed RCT. The 7 domains are: 1) confounding 2) Selection of participants 3) Classification of interventions 4) Deviations from intended interventions 5) Missing data 6) Measurement of outcomes 7) Selection of reported results. A n-RCT might therefore have been without major risks of bias compared to other studies of the same type, but compared to a RCT have issues, e.g., arising from randomization. All analyses were conducted at study level.

### Risk of bias across studies

Risk of bias across studies was analyzed using Robvis plot diagram [19] . Risk ratios (RR) were calculated to investigate trends.

## Results

### Study selection

The search resulted in 115 references from Cochrane library, 2063 from Embase, 534 from Medline and 13 from Cochrane trials. A total of 2715 references. No relevant ongoing studies were identified on clinicaltrials.gov nor was any material found on Opengrey.eu at the time of search (updated February 24th, 2021).

**Fig. 1:**
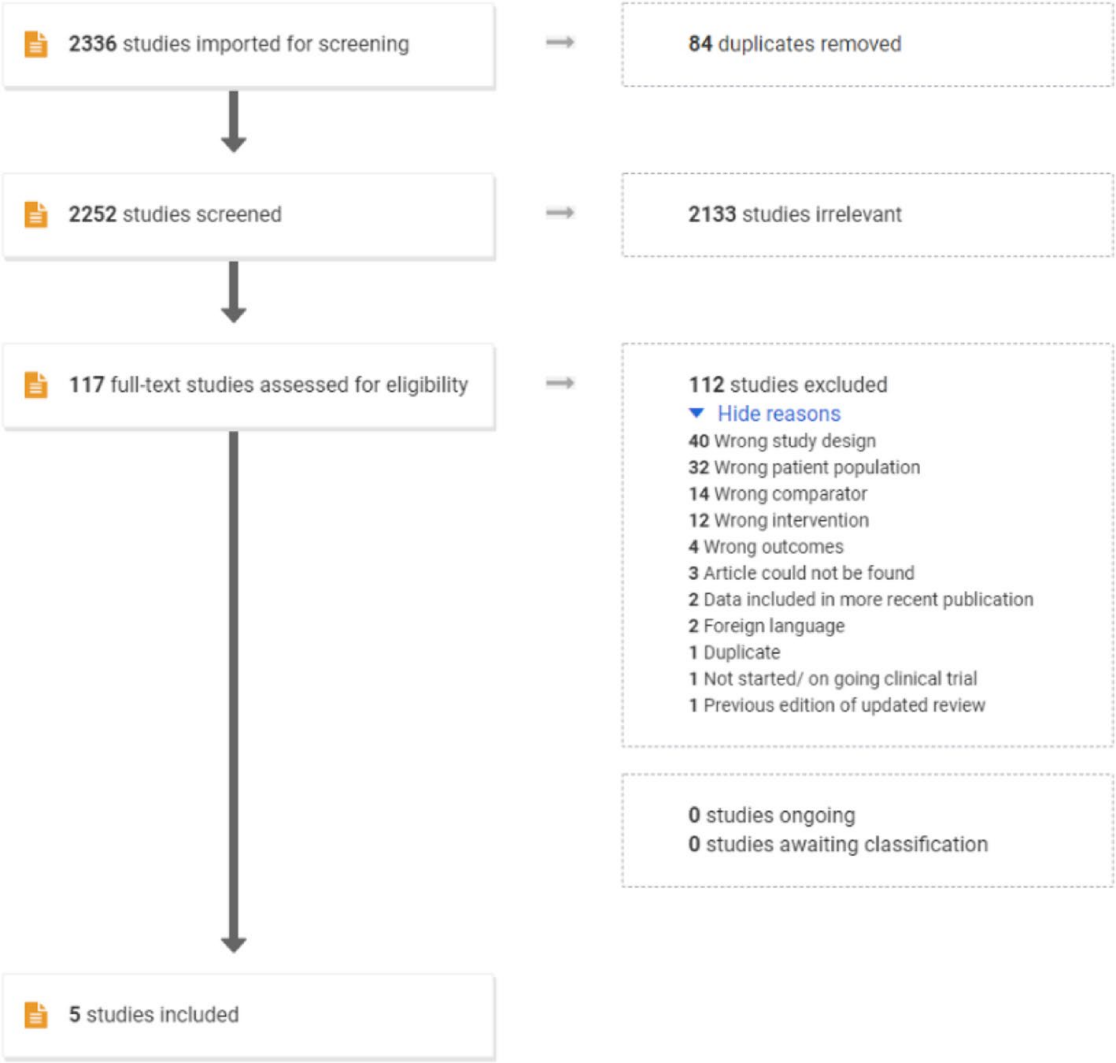
Original search flow from PRISMA

EndNote initially removed 379 duplicates. The remaining 2336 references were uploaded to Covidence for screening (Fig. 1). During the initial screening all titles were available in English. A total of 5 studies with 375 infants were included, 2 RCTs and 3 cohort studies (Table 1). The two RCTs have previously been included in a Cochrane review [7],

**Table 1:** Study characteristics of included studies

Study	Year	Methods	Participants	Interventions	Primary outcomes		Other effects of intervention
RCTs					Death	Surgery (including surgery for stricture, perforation, and re-NEC)	
Hansen [20]	1980	RCT Randomization from number table Blinded intervention	20 infants Overall mean GA: 35.2 weeks Overall mean BW: 2200 g	IV ampicillin 150 mg/kg/day ev. 6 h + IM gentamicin 7.5 mg/kg/day ev. 8 h ***Intervention Group***: + oral gentamicin through nasogastric tube 15 mg/kg/day ev. 4 h	Intervention: 1/10 Control:2/10	Intervention: 1/10 Control: 4/10	Intestinal perforation Intestinal stricture Peritonitis
Faix [21]	1988	RCT Randomization by sealed envelopes Unclear information about blinding	42 infants Overall mean GA: 29,4 weeks Overall mean BW 1300 g	IV ampicillin (< 7 days old 100 mg/kg/day ev. 12 h. > 7 days old 150 mg/kg/day) ev. 8 h + IV gentamicin (< 34 weeks GA 5.0 mg/kg initially then 2.5 mg/kg/day, > 34 weeks GA and < 7 days of age 5.0 mg/kg/day ev. 12 h, > 34 weeks of GA and > 7 days of age 7.5 mg/kg/d ev. 8 h. ***Intervention group*:** + IV clindamycin 20 mg/kg/day ev. 8 h	Intervention: 4/20 Control: 4/22	Intervention: 6/20 Control: 4/22	Bowel necrosis Strictures Time to successful reinstitution of enteral feeds Time to resolution of intestinal pneumatosis
**Cohorte studies**							
Vermeylen [22]	1985	Prospective cohort All children admitted during 1978–84 included	77 infants, (Group 1 included 1978–80 = 22 infants. Group 2 included after 1980 = 55 patients). Overall mean GA: 34 weeks Overall mean BW: 2005 g	IV ampicillin + gentamycin + enteral gentamycin 10–15 mg/kg/day ev. 4 h ***Intervention group:*** IV ampicillin + aminoglycoside + IV metronidazole booster 15 mg/kg followed by 7,5–10 mg ev. 12 h.	Intervention: 8/53 Control:7/22	Intervention: 17/24 Control: 4/22	Length of hospitalization
Scheifele [10]	1987	Prospective cohort All children admitted during August 1982–May 1985 included	90 infants (1982–83 = 46 infants included, from 1984 to 85 = 44 included infants) Overall mean BW: 1904 g GA?	1982–83: IV ampicillin 100 mg/kg/day + IV gentamicin 5–7.5 mg/kg/day. If age < 7d ev. 12 h, other ev. 8 h. 1984–85 ***Intervention group:*** IV cefotaxime 150 mg/kg/day + IV vancomycin 30–35 mg/kg/day. If age < 7d ev. 12 h, other ev. 8 h.	Intervention: 0/44 Control: 5/46	Intervention: 13/44 Control: 15/46	Peritonitis Intestinal perforation Thrombocytopenia Recurrent NEC Intestinal strictures
Luo [23]	2015	Retrospective cohort Infants treated from Jan 2008 to March 2015. Controls were chosen using PS-matching	146 infants (73 intervention group) Overall mean GA 38.86 Overall mean BW 2909 g	All infants received a combination of broad-spectrum antibiotics such as: semisynthetic penicillin, cephalosporin, carbapenems, vanco-mycin. *Intervention*: IV metronidazole 15 mg/kg/day	Intervention: 9/73 Control:7/73	Intervention: 11/73 Control: 6/73	sepsis blood transfusion Need for nasogastric suction

All included infants had radiological evidence of necrotizing enterocolitis (NEC). *Abbreviations*: *RCT* Randomized controlled trial, *IM* Intramuscular. *IV* Intravenous. *PS* Propensity score, *ev.* Every

On September 12th, 2020, the search was repeated. A total of 264 new references was added (Endnote initially removed 2 duplicates). The new references were screened by the author EMG, and none fulfilled the inclusion criteria.

#### Reasons for exclusion of studies

Several references were excluded due to population or study design not matching the inclusion criteria (tagged as wrong population in Covidence; etc. as it appears from Fig. 1); either because they included infants without confirmed NEC (studies investigating prophylaxis) or investigated the risk of developing NEC by maternal administration of antibiotics during pregnancy. The studies excluded for wrong study design was reviews or animal studies. Wrong comparator/intervention was used when studies switched between mixed treatment as this could be a possible confounder for the primary outcome [4].

### Included studies

None of the studies investigated a specific postoperative antibiotic regimen in children undergoing surgery. All infants continued the study specific treatment they had received before surgery.

### Results of bias in individual studies

**Fig. 2:**
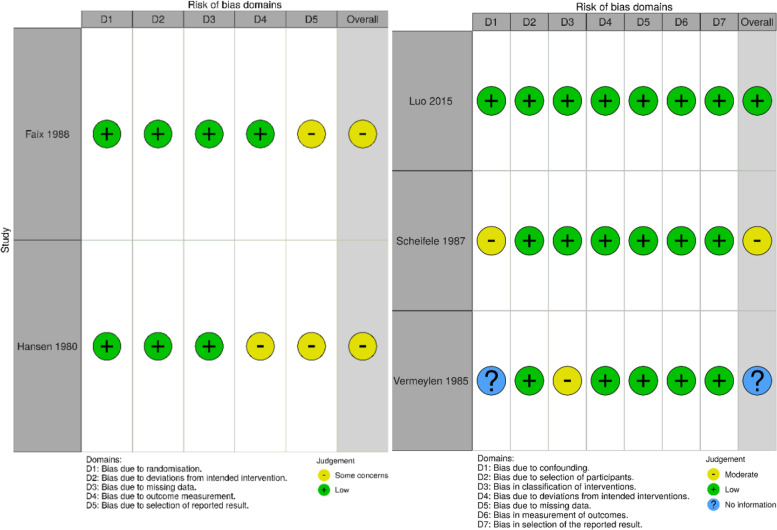
Traffic light plots for individual risk of bias in the included studies. Left: RCT, Right: n-RCT. Both plots were created using Robvis 19. Green indicates low risk of bias, yellow some concern and blue no information

Both of the included RCTs [20, 21]) raised some concern for bias in domain 5 (bias due to selection of reported result) as shown in Fig. 2. No pre-specified plan for study analysis was available and outcome measures were not presented in advance according to the criteria stated in Rob2 [17]. For the study by Hansen et al. [20] domain 4 gave rise to some concern for bias because the outcome assessors role in the study was unclear. No meta-analysis of the two RCTs was carried out because of too different treatment regimens to obtain reliable data.

As for the cohort studies, one study [23] was performed in a methodological manner comparable to a RCT. Two studies [10, 22] had one or more areas that gave reason to concern for bias.

In the study by Vermeylen et al. [22] information about statistical analysis of differences between the two groups compared were missing (domain 1). Scheifele et al. [10] provided well analyzed information about possible confounders, but according to standards specified by the ROBINS-I [18] tool it cannot be compared to a well performed RCT.

### Risk of bias across studies

All the cohort studies [10, 22, 23] had a low risk of bias regarding bias due to deviation from intended intervention (D4), missing data (D5), measurement of outcomes (D6) and selection of reported results (D7). Only the study by Luo et al. [23] could receive a low risk of bias equal to a well performed RCT.

**Fig. 3:**
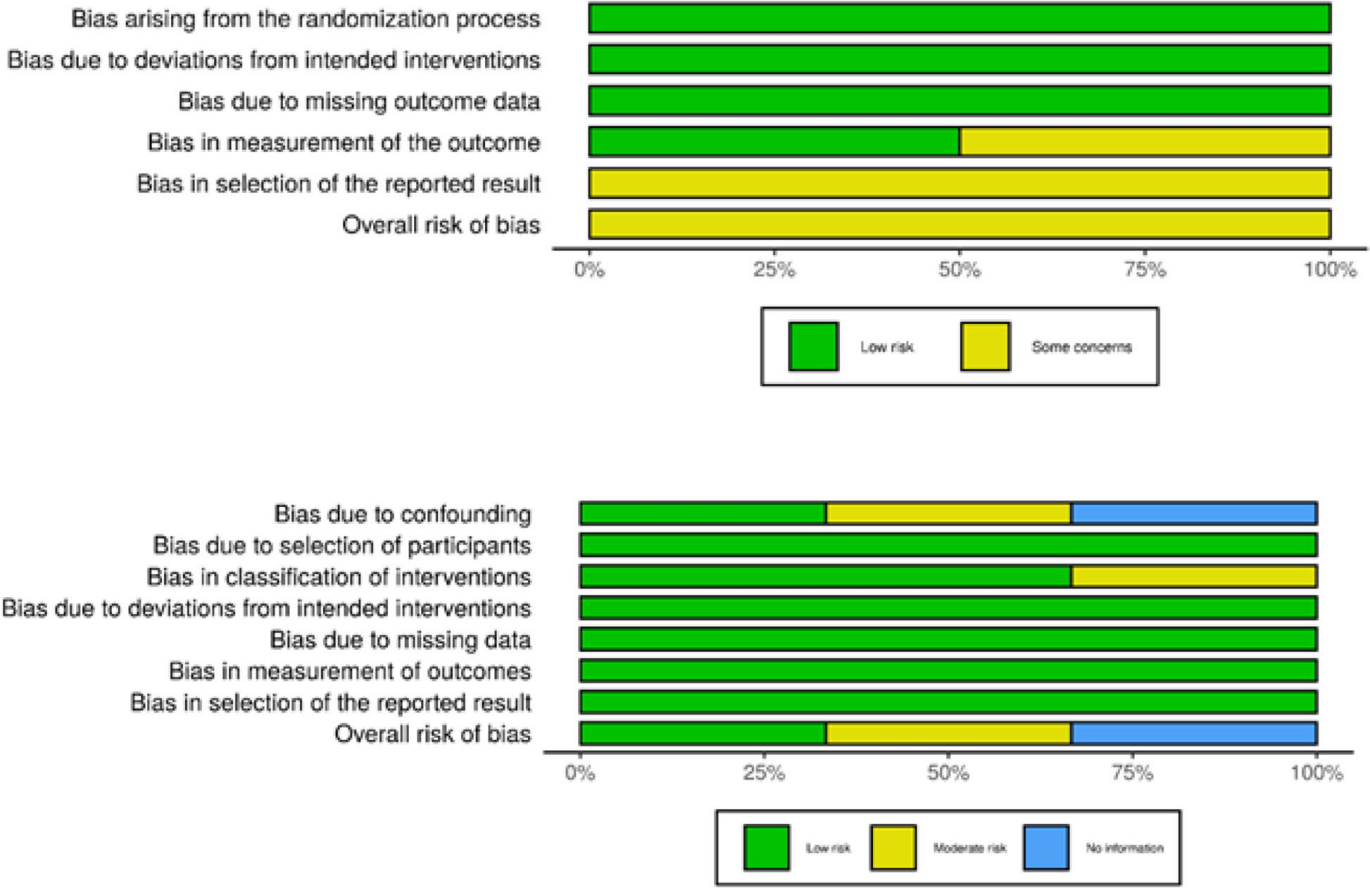
Summarized bias across studies. Top panel for RCT, lower panel for non-RCT. The plots were created using Robvis [19]

With the limited number of studies and difference in study setup it was decided not to calculate the weight of each individual study. Only one study was judged to be of overall low risk of bias [23], and it was decided there was no substance for a generalization (Fig. 3). No synthesis of studies was conducted due to the clinical heterogeneity of the studies (Table 1).

### Primary outcomes: death and surgery

For both the RCTs, the mortality rate in the intervention group varied from 10 to 20% and 18–20% in the control group with no significant differences. The values for surgery were 10–30% and 18–40%, respectively, and with no significant difference. In the three cohort studies the mortality rate varied from 0 to 15% in the intervention group and 10–32% in the control group. The values for surgery were 15–71% and 8–33%, respectively. The only significant difference found was in the surgical rate in the study by Vermeylen et al. [22] with the lowest rate in the group who received metronidazole but there was no difference in the mortality rates.

In the study by Luo et al. [23] 17 infants deteriorated from Bell’s stage II to III in a population of 143 infants. Eleven of these had received metronidazole and 6 had not. There was a lower mortality rate in the group receiving metronidazole, but the difference was not statistically significant.

Two studies specified the number of deaths for Bell’s stage III NEC. Vermeylen et al. [22] reported 5/30 deaths in the intervention group (metronidazole), compared to 4/4 deaths in the control group. In the study by Luo et al. [23] 1/11 infants receiving metronidazole died compared to 2/6 without metronidazole.

For the secondary outcomes no reliable data could be retrieved.

### Other effects of intervention

In the study by Faix et al. [21] there was no significant difference in the rate of bowel necrosis (perforation and gangrene) between the two groups (ampicillin+ gentamicin vs. ampicillin+gentamin + clindamycin). Hansen et al. [20] showed no significant differences regarding perforation, strictures, or death between the two groups of regimens (ampicillin+ gentamicin vs ampicillin+ gentamicin + oral gentamicin).

In the study by Scheifele et al. [10] children with a birthweight > 2200 g had better outcomes regardless of treatment. No infants in this group died, but had a similar risk of surgery as the children with a birthweight below 2200 g. For the group of infants with a BW < 2200 g, the group receiving standard treatment (ampicillin and gentamicin) had a significantly higher risk (*p* = 0.004) for surgery, peritonitis, strictures, but not for re-NEC (4/34 infants, *p* = 0.07 compared to the intervention with cefotaxime and vancomycin. No infants < 2200 g treated with cefotaxime/vancomycin died compared to 5/38 in the control group (*p* = 0.048).

## Discussion

The 5 included studies reported on 4 different treatment regimes. Although some few statistically significant differences emerged, and two studies [22, 23] showed a trend towards a positive effect from adding metronidazole to the regimen, we were not able to demonstrate consistent results to recommend neither the type of antibiotics, the route of administration or the duration of treatment. The information on the secondary outcome of death and NEC recurrence in those infants who underwent surgery were scarce and insufficient to achieve any conclusions.

A large variation in antibiotics combinations and duration among different institutions has been reported with 14 different combinations of antibiotics. Ampicillin+ gentamicin + metronidazole is reported as the most commonly used [24]. A study including patients with Bell’s type II and III NEC, found no combination of antibiotics to be superior from others and the duration of treatment had no significant implication of the treatment results.

During the literature search, a study from 2019 by Harutynyan et al. [15] was discovered. The study was not included because the number of deaths in Bell’s stage I disease could not be isolated. In addition, the study used antibiotics both as prophylaxis for infants in high risk of developing NEC, and treatment of infants with established NEC. A total of 200 infants were included from 2015 to 2018, where the intervention group (104 infants) were treated with a combination of oral gentamicin sulfate 15 mg/kg/day ev. 12 h + nystatin 10,000 IU/kg/day ev. 6 h and an orally administered symbiotic *(Lactobacillus rhamnosus)*. The control group received the standard treatment of empirical antibiotics (not specified). The overall mortality was 38/96 in the control group compared to 13/104 in the interventional group. For Bell’s stage III infants the numbers were 28/35 and 2/5, respectively. The difference was statistically significant. During the investigation period no infants underwent surgery in either group. It is unknown whether they received peritoneal drainage. A Cochrane review [9] from 2001 supports the finding that enteral antibiotics could have a prophylactic effect on the development of NEC, but raises concerns about unknown harms. Another Cochrane review from 2014 [25] concluded that the use of probiotics has an effect in preventing NEC in preterm infants. Although antibiotics is a core element in the treatment of NEC some studies seem to indicate antibiotics as a risk factor for developing NEC [26].

Ampicillin and gentamicin work synergistically and have created the base for many treatment regimens for NEC [5]. Hansen et al. [20] showed that the intervention group receiving oral gentamicin had significantly higher serum-gentamicin peak values compared to the control group (receiving only IV gentamicin) questioning the safety regarding known side effects of aminoglycosides.

One study investigating the effect of clindamycin was terminated prior to completion due to an increase in the frequency of intestinal stenosis [21]. A study by Autmizguine et al. [27] analyzing data from 2780 infants with NEC who were treated with or without antibiotics for anaerobic bacteria (clindamycin or metronidazole) showed that infants treated with the anaerobic antimicrobials developed more strictures (OR 1.73; 95% CI, 1.11–2,72). However, this was not significant when analyzed for clindamycin alone. Other studies [21, 23, 27] showed no effect of anaerobe antimicrobials in preventing progression of stage II NEC.

Piperacillin/tazobactam might be an alternative treatment in NEC. In a small study [28] with 27 very low birth weight infants there was a trend towards preventing progression of NEC. and no adverse events were reported.

The limitations of this review are studies of older date where methodology and reporting were of a different, less strict, and structured format. As a result, it was difficult to obtain equivalent information from all the included studies.

With the rising concerns about antibiotic resistance, efforts to find a treatment regimen with maximal effect is warranted, as is more knowledge on antibiotic treatment in the prevention and progression of NEC in addition to postoperative treatment. Another problem is the dose needed for a sufficient penetration of the antibiotic to the peritoneal cavity. The approved method with intraperitoneal microdialysis to measure antibiotic concentrations could be an option [29].

## Conclusion

No sufficient evidence was found for any recommendation on the choice of antibiotics, the route of administration or the duration in infants treated for Bell’s stage II and III NEC.

## Data Availability

The search string for this study can be found at https://www.crd.york.ac.uk/prospero/display_record.php?RecordID=162193. For data on analysis of risk of bias in the included studies, this can be available by request to the author EMG at ester.m.gill@gmail.com.

## Abbreviations

NEC: Necrotizing enterocolitis
BW: Birth weight.
GA: Gestational age
IM: Intramuscular
IV: Intravenous
PS: Propensity score
RCT: Randomized controlled trial
N-RCT: Non-randomized controlled trial
GI: Gastrointestinal
